# A Pilot Study Exploring the Optimization of Warm-Up Strategies: Modern Cognitive Warm-Up with Open-Skill Demands vs. Traditional Closed-Skill Warm-Up in Basketball

**DOI:** 10.3390/s25113279

**Published:** 2025-05-23

**Authors:** Asaf Shalom, Roni Gottlieb, Ido Shalom, Guy Gafni, Aviad Levy, Julio Calleja-Gonzalez

**Affiliations:** 1 Department of Physical Education, The Research Center for Sports and Physical Activity, Tel Hai College, Upper Galilee 12208, Israel; 2 Wingate Institute, The Academic College Levinsky-Wingate, Wingate Campus, Netanya 4290200, Israel; ronigot23@gmail.com; 3 Ort Magshimim High School, Nahariya 44221, Israel; idoshalom90@gmail.com; 4 Hapoel Jerusalem Basketball Club, 1st Division Israeli League, Jerusalem 94581, Israel; proswin1@gmail.com; 5 Department of Coaching Science, Lithuanian Sports University, Sporto Str. 6., LT-44221 Kaunas, Lithuania; aviadlevi86@gmail.com; 6 Department of Physical Education and Sports, Faculty of Education and Sport, University of the Basque Country, UPV/EHU, 01007 Vitoria-Gasteiz, Spain

**Keywords:** innovation in sports science, basketball, explosive power, warm-up, cognitive-motor dual-tasking, advanced sensor-based technology

## Abstract

Warm-up protocols are essential in high-intensity sports such as basketball, in which explosive power and rapid decision-making are critical for performance. This study examined the immediate effects of a modern cognitive warm-up, incorporating open-skill demands and cognitive-motor dual tasks, compared to a traditional closed-skill warm-up in youth basketball players. Twelve male players (ages 15–16) from an elite Israeli youth basketball club participated in the study and performed performance assessments post-warm-up. Sprint performance was evaluated using a closed-skill test (CST: 5-m and 10-m sprints without external stimuli) and an open-skill test (OST: 5-m and 10-m sprints with a reaction-based stimulus). The modern cognitive warm-up integrated advanced sports technology, and all performance assessments were conducted using reliable measurement technologies. The results demonstrated that the modern cognitive warm-up significantly enhanced sprint performance in both OST (*p* < 0.01) and CST (*p* < 0.05 for 5 m sprint), with no significant difference in the 10 m CST. Reaction times were also significantly improved (*p* < 0.01), emphasizing the effectiveness of cognitive warm-ups in enhancing perceptual-motor readiness. These findings suggest that integrating cognitive-motor dual tasks and open-skill elements into warm-up routines may enhance both readiness and focus for explosive performance, as well as improve players’ reactive abilities.

## 1. Introduction

In light of the global popularity of basketball, players are committed to exerting substantial efforts to achieve elite performance in both domestic and international basketball competitions [1,2]. Examining the physical and cognitive requirements of basketball can serve as a foundation for developing effective training strategies [3,4,5,6,7,8,9]. Basketball is an intermittent sport in which repeated high-intensity explosive actions including jumps, accelerations, decelerations, and changes of direction and pace are performed during practices and games [2]. In addition, basketball as an open-skill team sport requires cognitive and decision-making processing so that players can react to dynamically changing, unpredictable situations and conditions [9]. Thus, the development of psychomotor components such as agility, reaction time, coordination, balance, and kinesthesia is crucial for enhancing basketball players’ performance [9,10,11]. This ability to perform high-intensity explosive power actions combined with a demand for cognitive abilities is critical for basketball players’ success [7,9,12].

Given the high demand for this type of action, warm-up routines constitute indispensable components of athletic preparation, particularly in the context of high-intensity movement [13,14]. Warm-ups also serve as pedagogical strategies [15] prior to engaging in specific physical exertions, aimed at achieving an optimal state of psychophysical readiness [13,16]. These established practices are widely embraced by coaches and trainers, underscoring their recognized efficacy for enhancing performance and reducing injury risk [14,17]. Therefore, the preparatory exercises targeting specific movement patterns serve to reinforce both technical and physical skills [1]. Emotional adaptation is fortified through varying levels of psychological concentration [2,18]. Injury prevention is facilitated through warm-up routines, as multi-component exercises activate proprioceptive mechanisms, enhance tissue elasticity, reduce muscle viscosity, and promote strength and explosive power [14,19]. A traditional basketball warm-up routine for training and games can vary depending on factors such as the level of play, individual preferences, and specific goals. However, a typical basketball warm-up routine usually lasts around 20 min and includes general cardiovascular activation exercises, followed by dynamic stretching and, finally, a series of specific movement patterns at high tempo [3,5], in order to enhance explosive power, a significant ability in basketball [1,2,20,21].

Therefore, researchers and strength and conditioning coaches invest in building training methods to develop explosive power [3,5]. Numerous training methodologies have emerged for developing such power. For example, complex training and contrast training represent effective strategies for enhancing athletic performance by capitalizing on the acute effects of Post-Activation Potentiation (PAP) and neuromuscular facilitation [22,23,24,25,26]. Additionally, vertical and horizontal plyometric training represent complementary modalities for enhancing athletic performance and functional capacity by utilizing the stretch-shortening cycle (SSC) [3,27,28]. Modern methods for high explosive power performance have been developed in recent years. For example, multi-component training (MCT) offers a comprehensive and integrated approach to physical conditioning that addresses the diverse fitness needs of individuals across various contexts. By concurrently targeting strength, endurance, flexibility, and coordination, MCT provides a holistic strategy for optimizing athletic performance [9,29]. Additionally, cognitive-motor dual-task training (CMDT) offers a novel and effective approach to physical conditioning that integrates cognitive tasks with motor skill development to enhance overall performance outcomes [7,12]. By addressing the interconnectedness of motor skills and cognitive functions, CMDT provides a holistic strategy for optimizing cognitive function and athletic performance [7,9]. Extensive evidence indicates that this form of training is superior to and more suitable than sequential training methods [30,31]. Previous studies reported high significance for the CMDT training method for specific performance of explosive power combined with cognitive demands by basketball players [7,8,32]. Moreover, this training method was found to be highly significant in a five-week training program period when combined with MCT [9].

The main aim of this pilot study was to examine the potential differences between a modern cognitive warm-up that integrates open-skill demands, and a traditional warm-up that incorporates closed-skill demands, in relation to the specific and unique explosive performance of basketball players. This study addresses a notable gap in the literature, as limited empirical research has directly examined the acute effects of the differences between these two types of warm-ups on sport-specific performance among youth basketball players. Specifically, this study focused on the explosive performance of basketball players in short, high-quality linear sprints, similar to the sprinting distances required in basketball gameplay [2,20]. In addition, differences in reaction time were also taken into account and measured, as will be explained and described in Section 2. To the best of our knowledge, this study is among the pioneering investigations to examine and compare these types of warm-ups, with an in-depth focus on basketball. The research hypothesis is that the modern cognitive warm-up, based on open-skill demands and integrated with the use of sensor technology, will be more effective than a traditional warm-up based on closed-skill demands in improving performance, particularly in tasks that require open-skill execution. It is also expected that the modern warm-up will significantly enhance players’ readiness and reduce their reaction time.

## 2. Methodology

### 2.1. Participants

This study initially included 15 male basketball players aged 15–16 (mean age: 15.6 ± 0.4 years; body mass: 71.2 ± 5.1 kg; height: 179.3 ± 4.0 cm; and body fat: 11.4 ± 3.1%), who are all members of an elite youth league club in Israel, actively participating in professional training and competitions for at least seven consecutive years. Due to injury and illness, three players withdrew during the study, resulting in a final sample of 12 participants. Their weekly routine during the game season included five basketball practices, two fitness practices, and one league game. As the study was carried out during the preparation period, league games were not part of their weekly routine. Four inclusion criteria were applied in this study. Each participant (a) participated in at least 90% of the weekly training sessions during the preseason, the period in which the present study was carried out; (b) regularly participated in the previous season; (c) did not incur any injuries, were not in any pain, and were not taking any medication; (d) had a clean bill of health. All tests were carried out on the basketball court where the participants regularly practiced and played, to ensure familiarity with the testing environment. The participants wore basketball shoes and appropriate sportswear. The parents of all participants gave their informed consent before the study began, in accordance with the Declaration of Helsinki [33] after approval by the local ethical committee of the Levinsky-Wingate Academic College (Reference number: 435, 20 February 2024).

### 2.2. Procedure

To test the differences between the two types of warm-ups, participants were asked to alternate between the two types of warm-ups at 48-h intervals and under identical conditions. Both types of warm-ups included the same general component, including five minutes of exercises, consisting of dribbling and ending with a lay-up movement toward the basket, two and a half minutes on each side. This was followed by five minutes of dynamic stretching. The second part was more specific and reflected the differences between the two types of warm-ups. All 12 participants in the study experienced both types of warm-ups. The research team determined that participants would first perform the modern cognitive warm-up, which is based on open-skill movements, followed by the traditional warm-up, which is based on closed-skill movements, after a 48-h interval. This sequencing was intentionally designed to enhance the reliability and validity of the findings, ensuring a more robust assessment of the potential effects of the cognitive warm-up in accordance with the study’s hypothesis. By structuring the warm-up order in this manner, the researchers aimed to better isolate the impact of open-skill cognitive preparation on subsequent performance outcomes while minimizing potential learning or adaptation biases. In light of this, detailed explanations regarding the types of warm-ups are provided in Section 2.6 and Section 2.7. This addition ensures greater clarity and strengthens the reproducibility of the procedure.

### 2.3. Physical Tests

The traditional warm-up is based on closed skills and consists of controlled, pre-planned movements tailored to the specific demands of basketball, as compared to the modern cognitive warm-up, which is based on open skills and integrates dynamic, unpredictable tasks monitored by advanced technology. To assess the differences between the two approaches, a specialized test tailored for basketball players was conducted [1,2,9]. All participants performed a 5- and 10-m sprint test to evaluate sprint performance, explosive power, and reaction time [9,34]. This test was conducted in two variations on the same day with a recovery time of 5 min. All tests were initiated from a standardized standing start, with feet shoulder-width apart and the lead foot positioned behind the starting line. The starting position was monitored and verified by the researchers to ensure consistency across all participants.

To systematically differentiate between the testing conditions, a standardized notation system was implemented. Each abbreviation consists of three components, where each letter signifies a specific aspect of the test, as follows:

- First letter (T or C) represents the type of warm-up performed, as follows:
  ○ T—Traditional warm-up;
  ○ C—Cognitive warm-up.
- Second letter (C or O) indicates the skill condition, as follows:
  ○ C—Closed-skill test (CST);
  ○ O—Open-skill test (OST).
- Third component (5 M, 10 M, RT) denotes the measured parameter, as follows:
  ○ 5M/10M—Sprint distances (5 m or 10 m);
  ○ RT—Reaction time measurement.

For example, T-C-5M refers to the Traditional warm-up (T) in a Closed-skill test (C), measuring the 5-m sprint (5M). Likewise, C-O-RT refers to the Cognitive warm-up (C) in an Open-skill test (O), measuring Reaction Time (RT).

This notation system, as well as Table 1 (which will be presented below), is essential for understanding this section.

By employing this standardized framework, this study ensures methodological clarity and facilitates precise performance comparisons across experimental conditions.

**(a) Closed-Skill Test (CST):** Participants performed the test freely without specific cognitive components, starting at their own pace. Time was measured at 5 m (T-C-5M, C-C-5M) and 10 m (T-C-10M, C-C-10M) within a single sprint. Each participant completed two attempts, and the best result was recorded [1,2].

**(b) Open-Skill Test (OST):** This variation required participants to react to stimuli and make decisions. Unlike the CST, where participants performed the tasks freely, this test required them to spring in response to a visual cue. A Witty-SEM device displayed a red-light countdown (3-2-1), which turned black before a green light appeared at random intervals (1–5 s), signaling the start [9]. Sprint times were recorded at 5 m (T-O-5M, C-O-5M) and 10 m (T-O-10M, C-O-10M), following the established notation system.

Additionally, reaction time (RT) was measured separately, assessing the players’ ability to initiate sprinting in response to an external stimulus. All reaction time tests (RT) were conducted exclusively under the OST condition, as these tests were specifically designed to measure responses to external stimuli [9]. In this study, the measurement of reaction time (RT) was defined as the time elapsed from the onset of the external stimulus to the execution of the first propulsion movement, which was determined as the first explosive step within the initial 50 cm of the sprint, measured before the starting line, as will be described in Section 2.4. This setup aligns with open-skill environments, where participants must react dynamically to unpredictable visual cues. This assessment was conducted to evaluate the influence of reaction time as an additional indicator in tests requiring open-skill proficiency. Furthermore, it is important to note that standard reaction time measurement is typically shorter and faster than the reaction time defined and measured in this study [35].

### 2.4. Use of the Measuring Tool

The sprint-based tests comparing the effects of traditional warm-ups vs. modern cognitive warm-ups were conducted using the MicroGate system (Bolzano, Italy), which features a high-precision receiver base designed to detect, capture, and transmit anticipatory movements with exceptional accuracy [2,8,9]. In the CST condition, data were recorded using six Witty-GATE photocells, positioned in parallel lines to ensure accurate sprint time measurement. The 5-m and 10-m sprint results were obtained within a single sprint execution, allowing for simultaneous evaluation of short-distance acceleration and extended sprint performance [2,3,36,37]. In the OST condition, the same photocells were used for consistency, but an additional Witty-SEM device was placed in front of participants [9,38], providing a visual stimulus to initiate the sprint.

During the tests, participants were required to begin all sprints 50 cm before the starting line, in accordance with established and widely accepted methodological standards in the sports science literature [39]. This ensured uniformity and consistency across trials. Each athlete completed two trials, with the faster recorded time used for further analysis. This added distance was selected to enhance the assessment of reaction time, particularly in open-skill sprint tests, where participants were required to react to an external stimulus before initiating movement. Additionally, the controlled and standardized setup minimized external influences that could impact the reaction phase and acceleration dynamics. By maintaining a consistent starting position, this approach optimized the test results, allowing for a precise and direct comparison of reaction time, as defined in this study, and all types of sprint tests between the traditional and modern cognitive warm-up protocols.

Both the Witty-GATE and Witty-SEM systems utilize advanced sensor technology to detect motion onset with high temporal precision, ensuring reliable measurement of sprint times and reaction delays. The integration of these sensors minimizes human error and enhances the objectivity of performance assessments. In the open-skill tests (OST), both systems were used synchronously, with Witty-GATE recording sprint times and Witty-SEM providing real-time visual stimuli. This synchronized use of sensor technology ensured precise alignment between reaction initiation and movement execution, allowing for a comprehensive evaluation of response efficiency in dynamic conditions [2,9,36,37,38]. The technology used in this study applied existing, well-established tools innovatively to examine the effects of cognitive warm-up in basketball.

### 2.5. Warm-Up

The modern cognitive warm-up performed by the participants included three stations of open-skill tasks, while the traditional warm-up included three stations of closed-skill tasks. A set for any of the tasks lasted up to 10 s. Recovery time after each task was 3 min [2,3].

### 2.6. Traditional Warm-Up Protocol

#### 2.6.1. Cones in Space

Six cones were placed in the center of the half court: four cones in the shape of a square four meters from each other, and two more cones were placed in the center of the square, one meter from each other. While dribbling, participants were required to run within the area and touch the six cones in whatever order they wished. After each touch of the cone chosen by the participants, they had to return to the initially defined starting point (the center of the square). Although participants freely chose the cones they touched, they could not return to the same cone in two consecutive repetitions. After completing 3 repetitions of the task, they ended with a “layup” move toward the basket. The participants started the task in the center of the square and were required to perform the task at a high tempo.

#### 2.6.2. Cones in a Line

Five cones were arranged in a row at one-meter intervals, and participants stood one and a half meters in front of the row of cones. They were required to accelerate promptly, navigate around one cone of their choice, and return to the predetermined starting point. In this task as well, participants freely chose the cone they would circle, and could not return to the same cone in two consecutive repetitions. Participants chose when to initiate the task and were required to perform the task at a high tempo. They were required to complete four repetitions of the exercise at this station.

#### 2.6.3. Cut and Sprint

Participants were positioned at a designated spot, encircled by four cones arranged in a square formation, with each cone spaced two meters apart. Participants chose when to initiate the task. They were instructed to accelerate, execute a progressive sprint, and touch three cones of their choosing. Following each cone touch, they were required to return to the predefined starting point (the center of the square). In this task, participants had the freedom to select which cones they touched; however, they were instructed not to return to the same cone in two consecutive repetitions. At the end of the task, participants were required to perform a sprint of 10 m to a predetermined point.

All the tasks were monotonous, and participants completed three repetitions in every station. The cognitive warm-up also comprised the same three tasks as in the traditional warm-up, but it also included variables and tasks requiring open skills. As with the traditional warm-up, one set of any of the tasks lasted up to 10 s, and the recovery time after each task was 3 min [3,5].

### 2.7. Modern Cognitive Warm-Up Protocol

#### 2.7.1. On-Court Reaction While Dribbling

Using the BlazePod^TM^ system [40,41], six pods were placed in the center of the half-court. As in the traditional warm-up, pods were arranged in the shape of a square four meters from each other. Another two pods were placed in the center of the square, one meter from each other. Throughout the task, these pods displayed a range of colors at random intervals. While dribbling, participants were required to react promptly to the pre-assigned colors by reaching the corresponding pod (right hand for blue, left hand for red), all while discriminating between the target colors and distractor colors (purple, yellow, pink, green). This exercise challenged participants to respond to environmental distractions in a dynamic space. Following each action, participants were required to return to the designated starting point. The task concluded with a layup move toward the basket after a series of 3 repetitions at a high tempo. The BlazePod^TM^ system provided a scientifically validated method to enhance cognitive-motor processing by requiring real-time decision-making under reactive conditions. The use of light-based stimuli ensured an unpredictable training environment, mimicking real-game scenarios where players must respond to unexpected cues under pressure. By employing sensor-based technology, this warm-up method aimed to improve visual–motor coordination, reaction speed, and decision-making efficiency in a sport-specific setting [40].

#### 2.7.2. Human Mirror Reaction Station

Two participants stood in two different positions, each surrounded by four cones. The participant in the first position was the leader and had to move quickly and touch one of the four cones of his choice, and then return to the designated starting point. The participant in the second position had to react by anticipating and responding quickly, by moving to the corresponding cone in his own position—mirroring the first participant’s choice. For example, if the first moved to the front cone, the second had to move to the front cone in his position. Participants were required to perform four repetitions of this task at a high tempo, after which they switched roles.

#### 2.7.3. Crazy Ball Drill

Participants were instructed to throw a reaction ball (referred to as a “crazy ball”) vertically to head height from a predetermined starting position and then react by catching the ball after it bounced once on the court surface. After each successful catch, participants were required to promptly return to their initial starting position. After completing three repetitions of this task, participants were required to execute a rapid 10-m sprint toward a designated cone.

### 2.8. Statistical Analysis

To ensure the robustness of the statistical evaluation, we conducted descriptive statistical analyses, a paired t-test, and the Wilcoxon matched-pairs test. Data are reported as mean ± standard deviation (SD) and coefficient of variation (CV). A priori power analysis was conducted using G*Power to determine the appropriate sample size. Post-hoc power analysis was performed for the five Wilcoxon paired comparisons. Cohen’s d effect sizes were used to assess the magnitude of the difference and were interpreted as follows: d < 0.20 was considered trivial, 0.2–0.49 small, 0.5–0.79 moderate, 0.80–1.0 large, and d > 1.0 very large. Significance was set at *p* < 0.05 for all statistical tests, and analyses were conducted using JASP (version 0.19.3).

## 3. Results

The results of the physical performance tests following the two types of warm-ups are presented in Table 1. This table provides a systematic summary of the sprint and reaction time assessments conducted in this study, with further data to be presented later in this section.

The coefficient of variation (CV) remained below 5%, demonstrating consistency in participant performance. All OST tests (T-O-5M vs. C-O-5M, T-O-10M vs. C-O-10M, T-O-RT vs. C-O-RT) exhibited large effect sizes and achieved relatively high statistical power (~0.86), indicating a robust and consistent impact of this warm-up method.

In contrast, CST (T-C-5M vs. C-C-5M) demonstrated a moderate effect size with moderate statistical power (~0.57–0.69), suggesting a meaningful but less pronounced effect compared to OST. However, CST (T-C-10M vs. C-C-10M) showed a small effect size with lower statistical power (~0.09–0.29), limiting its ability to detect significant differences. The relevant performance data and statistical outcomes are summarized in Table 1.

**Figure 1:**
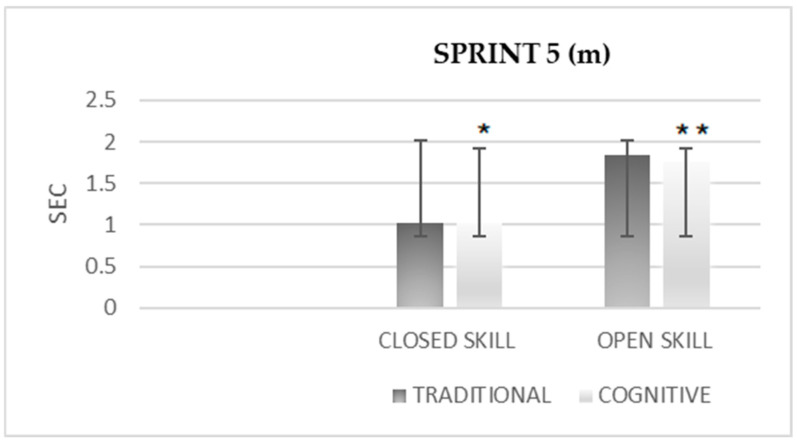
The difference in 5-m sprint after traditional vs. cognitive warm-up. * *p* < 0.01 ** *p* < 0.01.

The difference in the 5-m sprint (Figure 1) after the cognitive warm-up was significantly greater than after the traditional warm-up in both the closed-skill and open-skill conditions (M = 1.03 ± 0.028 vs. M = 1.022 ± 0.025, M = 1.845 ± 0.119 vs. M = 1.761 ± 0.094, respectively).

**Figure 2:**
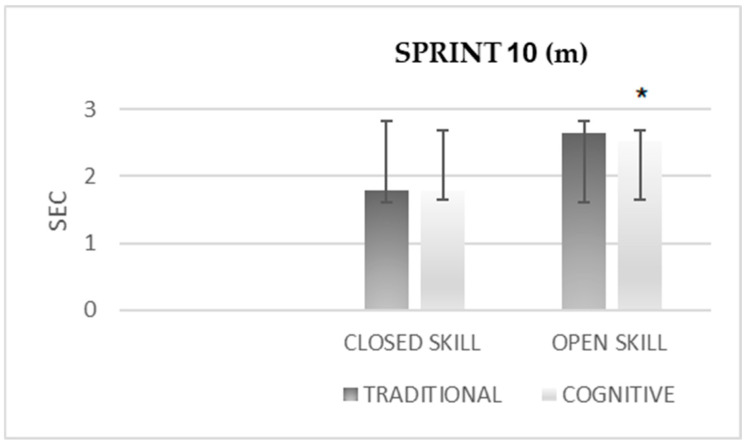
The difference in 10-m sprint after traditional vs. cognitive warm-up. * *p* < 0.01.

The difference in the 10-m sprint (Figure 2) after cognitive warm-up was significantly greater than after the traditional warm-up in the open-skill condition (M = 2.535 ± 0.126 vs. M = 2.656 ± 0.129). In closed skills, no significant differences were observed after the cognitive warm-up compared to the traditional warm-up.

**Figure 3:**
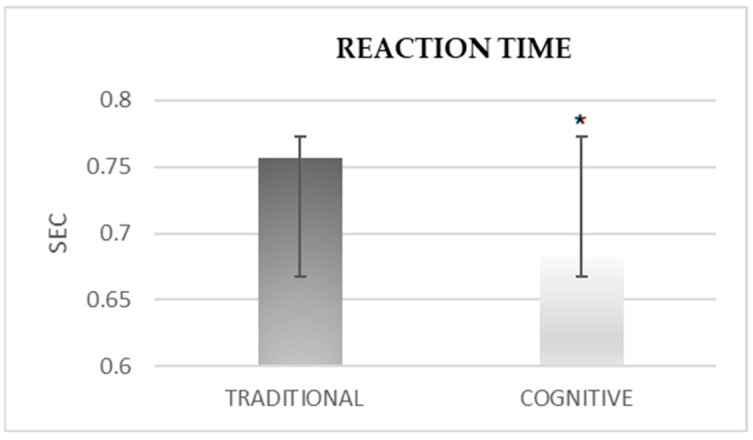
The difference in reaction time after traditional vs. cognitive warm-up in open-skill tests. * *p* < 0.01.

The difference in reaction time (Figure 3) after the cognitive warm-up was significantly greater than after the traditional warm-up in the open-skill condition (M = 0.683 ± 0.082 vs. M = 0.757 ± 0.1).

## 4. Discussion

In the present pilot study, we examined the potential effects of implementing an innovative cognitive warm-up approach, based on Modern Cognitive Motor Dual-Tasking principles and designed around open-skill demands, which could be more effective than a traditional warm-up relying on closed-skill demands. Previous studies investigating the impact of short-term training interventions incorporating CMDT demonstrated significant performance improvements in basketball players [8,9]. Given the effectiveness and positive influence of these short-term training methods, which emphasize open-skill demands, this study aimed to develop and implement a modern cognitive warm-up based on open skills, structured within the CMDT framework, and to assess its effects on explosive power performance in young basketball players.

The results demonstrated that the modern cognitive warm-up was significantly more effective in enhancing performance compared to the traditional warm-up. Based on this study, preparing basketball players for explosive power performance using a warm-up that incorporates cognitive multi-tasking demands is statistically significant and highly effective. In the open-skill tests (OST), which required players to react to stimuli—specifically the 5 m and 10 m sprints—significant differences were observed between the modern cognitive warm-up and the traditional warm-up, with superior performances recorded following the modern cognitive warm-up (*p* < 0.01). Additionally, reaction time was analyzed in isolation between the two warm-up protocols, as detailed in Section 2.

The findings revealed that participants who underwent the modern cognitive warm-up demonstrated significantly faster reaction times compared to those who performed the traditional warm-up (*p* < 0.01), highlighting its effectiveness in enhancing perceptual-motor readiness. In the closed-skill test (CST), a significant advantage was also observed in the 5 m sprint (*p* < 0.05), whereas no significant differences emerged in the 10 m sprint. These results suggest that while the cognitive warm-up benefits tasks requiring reactive agility and stimulus-driven execution, its effect is less evident in longer, linear sprints without external stimuli. Although this advantage was statistically less pronounced compared to the open-skill sprint tests and reaction time assessments, it remains an important finding that warrants further discussion. This suggests that the benefits of the cognitive warm-up extend beyond stimulus–response tasks and may influence certain aspects of short-distance acceleration, even in closed-skill scenarios.

These findings point to the potential effectiveness of the cognitive warm-up approach introduced in this pilot study, which integrates diverse open-skill demands with advanced technology. The results indicate that this modern cognitive warm-up is more effective than traditional warm-up methods, particularly in tasks requiring explosive power with stimulus–response demands (open-skill tasks) and even in certain closed-skill sprint performances. These findings reinforce the importance of integrating cognitive components into warm-up routines, particularly in sports like basketball, where explosive power, rapid decision-making, and reaction speed are key determinants of success. While traditional warm-up protocols emphasize physiological preparation, this study highlights the potential for cognitive activation to enhance performance in both reactive and non-reactive sprinting tasks. The implications of these results will be further examined below, including the potential applications and limitations of cognitive-based warm-up strategies in competitive sports settings.

Expanding on the role of cognitive activation, research in other performance domains has also demonstrated its benefits. A previous study by Katada et al. [18] found that a warm-up incorporating guided imagery, or a combination of breathing, movement, and sensory awareness, significantly improved choral performance. In contrast, a warm-up based solely on breathing or stretching primarily enhanced subjective readiness without a significant objective impact on performance. These findings are relevant to the present study, as targeted and specific preparatory exercises enhance not only technical and physical skills but also emotional adaptation through psychological focus. The more tailored the warm-up is to the specific demands of the field, the more likely it is to optimize performance. Based on this study, it is plausible that integrating mental processing with physical preparation is essential not only for musicians but also for athletes—particularly ballgame players and basketball players—who must perform optimally in dynamic environments [18]. Accordingly, research on cognitive training suggests that enhancing attentional focus and decision-making processes can contribute to performance improvement.

Previous studies on cognitive training have demonstrated that CMDT-based interventions improve reaction speed, likely by enhancing attentional allocation and decision-making processes after sensory processing [9,42]. The neural basis of this effect has been identified in previous research as an increase in the P3 ERP component, which is associated with various post-perceptual cognitive functions, including decision-making [42]. This mechanism may have contributed to the improved performance in stimulus–response tasks in the present study. However, to establish the scientific validity of these findings, future studies should explore the specific impact of warm-ups on performance rather than solely focusing on training methodologies.

One of the key findings in the present study was the significant advantage observed in the 5 m sprint (CST category) following the modern cognitive warm-up. Interestingly, this finding was not initially considered in the research hypothesis. It could be attributed to the warm-up’s enhancement of concentration and focus in short, explosive, and precise movements. Conversely, in the 10 m closed-skill sprint within the same category, no significant differences were found between the warm-up methods, suggesting that cognitive warm-ups may be less relevant for longer sprint performances that do not require immediate decision-making or stimulus–response adaptation. Another possible explanation (OST category) for these findings is that a central aspect of the modern cognitive warm-up included cognitive preparation for a range of psychomotor abilities, such as rapid recognition and anticipation of relevant stimuli in dynamic situations. These skills are particularly crucial in basketball, where players must quickly process and respond to unpredictable in-game scenarios. The specific elements incorporated in the modern cognitive warm-up, which emphasize open skills, significantly contributed to the players’ readiness for performance. While the traditional warm-up involved movements relevant to basketball, the absence of open-skill demands—such as stimulus–response adaptation and decision-making—resulted in less effective preparation for performance, as demonstrated in this study.

Previous research on warm-up effects in ball games has primarily focused on warm-up duration, differences between stretching types, and injury prevention strategies, emphasizing that dynamic warm-ups are highly effective for preparing athletes for explosive actions [13,14,15,16,43]. However, to the best of the authors’ knowledge, only one study in the literature has directly compared an open-skill warm-up with a closed-skill warm-up in basketball players [44]. In the study by Gabbett et al. [44], 14 basketball players (6 males, 8 females, mean age of 16.3) were tested.

- **Open-skill warm-up:** Included drills requiring responses to unpredictable elements, such as dribbling and reacting to other players.
- **Closed-skill warm-up:** Consisted of predetermined movements, such as planned sprints and controlled jumps.

Participants were assessed in various basketball-relevant tests measuring explosive power demands, including sprint speed (5 m, 10 m, and 20 m), change-of-direction speed (*t*-Test), vertical jump, and reactive agility (response to examiner movement). The study found no significant differences (*p* > 0.05) between the two warm-up types across all performance measures. Even the open-skill warm-up did not significantly enhance reactive agility, contradicting the study’s initial hypothesis. Additionally, the closed-skill warm-up did not impair performance in open-skill tasks such as rapid responsiveness or change-of-direction movements. A key conclusion of that study was that reactive agility requires prolonged exposure to gameplay and that a short warm-up is insufficient to improve cognitive skills such as visual cue recognition and decision-making [44].

A recent study by Cerrillo-Sanchis et al. [45] further highlights the importance of sport-specific warm-up protocols by comparing the effects of a general injury-prevention warm-up designed for soccer players (FIFA 11+) with a basketball-specific warm-up (Basket-Up), which incorporates open-skill demands. Conducted on elite youth basketball players, the study evaluated immediate performance improvements across agility, sprint speed, and vertical jump. The findings revealed that while both warm-ups contributed to overall readiness, the basketball-specific Basket-Up protocol, which integrates cognitive and motor tasks under dynamic and unpredictable conditions, was significantly more effective in enhancing agility performance (*p* = 0.001). These results highlight the potential advantages of incorporating open-skill cognitive training in warm-ups tailored to the specific neuromuscular and perceptual demands of basketball. While traditional warm-ups, such as FIFA 11+, provide structured and controlled activation exercises, they may not fully prepare athletes for the decision-making and rapid motor adjustments required in high-intensity basketball scenarios. This aligns with the present study’s findings, reinforcing the notion that sport-specific cognitive-motor warm-ups may offer superior benefits for immediate performance enhancement in basketball.

To the best of our knowledge, the present study is among the few pioneering investigations that shed light on the efficacy of a modern cognitive warm-up based on open-skill demands in enhancing immediate basketball performance. This topic has not been extensively researched and requires further investigation. The recent study by Cerrillo-Sanchis et al. [45] also represents one of the early contributions to this field, demonstrating the positive effects of a warm-up protocol that incorporated open-skill elements. However, while their findings highlight the benefits of integrating cognitive-motor demands within warm-up routines, their study focused on a protocol not exclusively based on open-skill tasks, and the comparison was made with an effective warm-up protocol specifically designed for soccer players. In contrast, the present study introduces a highly specialized and innovative protocol that is structured around open-skill demands and incorporates advanced technology to optimize cognitive-motor engagement. Furthermore, the warm-up comparisons in this study were uniquely tailored to the demands of basketball, ensuring a sport-specific evaluation of different warm-up strategies.

The findings reinforce the growing body of evidence supporting cognitive-motor integration in pre-performance routines, particularly in sports that require rapid decision-making and reactive motor execution. Moreover, based on the results—particularly the findings in the OST category, which showed a highly significant improvement after the modern cognitive warm-up—it can be inferred that participants did not enhance their explosive power per se as a direct result of the warm-up. Instead, it was primarily the cognitive readiness that contributed to the improved performance enhancements in execution. Additionally, to the best of our knowledge, this study is the first to specifically examine reaction time capability, as defined in this study, in short sprints based on open-skill execution in basketball, comparing different warm-up protocols. These findings provide novel insights into the impact of an innovative modern cognitive warm-up vs. a traditional warm-up, as presented in this study, highlighting its potential contribution to enhancing reactive readiness and neuromotor performance in sports requiring rapid decision-making and dynamic motor execution.

Interestingly, the significant improvement observed in the 5 m closed-skill sprint test suggests that cognitive preparation may also play a role in enhancing short, high-quality performances even in closed-skill contexts. This raises important questions for future research, as further studies should explore the extent to which cognitive warm-ups impact closed-skill tasks and whether heightened mental readiness and focus contribute to improved execution in short-distance accelerations. While this remains an open area for investigation, the findings provide valuable insight into the broader implications of cognitive warm-up strategies, suggesting that cognitive priming may enhance performance not only in open-skill environments but also in select closed-skill tasks that require high levels of focus and readiness.

Recent innovative studies in the literature follow a similar line of thought. Active breaks have been suggested to support recovery and enhance performance, and motor-cognitive active breaks in particular have been shown to improve sport-related reactive agility. Performance improvements following active breaks appear to be primarily driven by enhanced cognitive functioning rather than physiological recovery, offering a potential advantage for athletes, especially in ball and team sports [46]. Additionally, research comparing motor-cognitive exercises and exergaming to computer-based brain training has found that motor-cognitive tasks elicit stronger brain activation and better transfer of learning, likely due to the integration of complex motor demands. Motor-cognitive tasks also show greater neural engagement and functional connectivity compared to purely cognitive tasks [47]. Together with previous findings, which also help to better understand the unique demands of basketball [2,46,47], this supports the relevance of the current study [9,48,49], whose main innovation lies in demonstrating the immediate positive effect of cognitive preparation on performance enhancement, specifically as optimal preparation for ball games and basketball.

One explanation for the implementation of the research hypothesis in the present study, compared to the study by Gabbett et al. [44], is that the modern cognitive warm-up in this study incorporated a variety of contemporary drills specifically designed to meet the demands of basketball, including the use of advanced technology, as described in the Methods Section. The components selected for examination in this study, such as explosive power and reaction time measured through the 5-m and 10-m sprint tests, have been extensively evaluated in previous studies and reported as highly relevant to basketball performance [2,3,5,8,9,47]. Additionally, the present study selected two key test categories conducted immediately post-warm-up, ensuring direct impact evaluation. This focused approach may have enhanced the effectiveness of the cognitive warm-up. Another key takeaway from this study is that the traditional warm-up was consistently less effective across all tested performance measures. This provides valuable insights for coaches considering the integration of a modern cognitive warm-up based on open skills, which proved highly effective in this study, as part of pre-game or pre-training routines. Optimizing warm-ups for specific training sessions where coaches aim to maximize players’ potential could be highly relevant depending on training objectives. Regarding game preparation, the demonstrated efficacy of the modern cognitive warm-up should be taken into account.

## 5. Study Limitations

This study provides valuable insights into the effectiveness of a cognitive warm-up based on open-skill demands; however, several limitations must be acknowledged. First, the small sample size (12 male basketball players) limits the generalizability of the findings to broader populations, including female athletes and players of varying skill levels. Recruiting larger athlete samples is inherently challenging, as many are unable to adhere to the required training and supplementation protocols. Additionally, the use of a convenient, non-probabilistic sampling method may introduce selection bias, potentially limiting the representativeness of the results. Another limitation is the lack of gender and age diversity, which constrains the ability to determine whether the observed effects apply uniformly across different demographic groups. Furthermore, this study did not account for position-specific variations among players, despite the distinct physiological and cognitive demands of guards, forwards, and centers. This study also focused primarily on sprint performance in open- and closed-skill tasks, without assessing other critical performance indicators such as vertical jump height and change-of-direction ability, which are essential components of basketball performance. Additionally, data collection was conducted exclusively during the preseason preparation period, without considering other phases of the competitive season. As a result, the long-term effects of cognitive warm-ups on neuromuscular adaptations and injury risk under competitive conditions remain unclear. Participants were not randomly assigned to groups, due to the considerations outlined in the Methods Section. The absence of randomization represents a methodological limitation that should be acknowledged. Reaction time was measured as the interval between the onset of the external stimulus and the initiation of the first propulsion movement, defined as the initial explosive step within the first 50 cm of the sprint, prior to crossing the starting line. This measurement has certain limitations, as the short distance may reduce the sensitivity of the assessment. Although efforts were made to ensure standardized starting positions across all participants, this aspect should still be considered a methodological constraint. Nonetheless, this variable was included in the study to provide an additional though not primary indication of the potential effects of cognitive warm-up, and was analyzed alongside other, more standardized performance tests presented in the study.

## 6. Future Research Directions

Further research should focus on integrating a broader range of performance tests in a structured and systematic manner to ensure an optimal research design for evaluating the full spectrum of warm-up effects. A comprehensive approach should include assessments of neuromuscular activation, cognitive load adaptations, and physiological responses to different warm-up protocols. Additionally, studies should explore sport-specific adaptations and the potential long-term benefits of open-skill cognitive-motor warm-ups across various competition levels and athlete demographics, focusing on how these warm-ups influence in-game decision-making, fatigue resistance, and injury prevention. Examining and comparing the effects of innovative warm-up routines across athletes from different sports could provide insights into the transferability and sport-specific effectiveness of cognitive-motor warm-ups. Further exploration should assess the impact of innovative open-skill-based warm-ups on a broader range of performance measures, including agility, reaction-based motor tasks, and sport-specific movements. Another important research direction is to assess open-skill cognitive warm-ups across multiple sessions, systematically separating different test categories across days or phases to control for performance variability due to accumulated fatigue or adaptation effects.

It is important to note that the current study did not include direct measurement of physiological responses during warm-ups; effects were evaluated based only on performance outcomes. Future studies should incorporate both physiological and neurological monitoring during warm-ups, using wearable sensors to track heart rate, muscle activation, or other physical indicators, and EEG-based measures to assess brain activity. Specifically, the P3 ERP component could provide valuable insights into attentional and cognitive processing during and after different warm-up protocols, enhancing the understanding of how warm-up methods influence both physical performance and cognitive function. Moreover, investigating the optimal duration and intensity of cognitive warm-ups, and their potential interactive effects with physical warm-up components, could improve their applicability in elite sports settings. By expanding research on cognitive-motor warm-ups, future studies can contribute to a deeper understanding of their mechanistic effects, enabling the development of evidence-based protocols tailored to high-performance sports demands. In addition, studies should investigate whether cognitive warm-up adaptations differ by age, gender, and playing position.

Finally, this pilot study introduced a modern cognitive warm-up integrating sensor-based technology, a reaction ball, and the anticipation of basketball-specific human movement patterns. Future studies should examine how effective sensor-based warm-ups are compared to other, more widely available methods used in the field. Future studies should aim to better understand how advanced technology can be applied in sports science, especially in using sensor-based equipment for performance measurement vs. types of warm-ups or training exercises.

## 7. Conclusions and Practical Recommendations

In the context of optimizing warm-up strategies, this study highlights that modern cognitive warm-ups based on open-skill demands may offer distinct advantages over traditional closed-skill warm-ups in basketball. The findings of this pilot study offer preliminary support for the potential effectiveness of a modern cognitive warm-up based on open-skill demands in enhancing explosive power performance in basketball players. Given the significant advantages observed in both open- and closed-skill sprint performance, integrating cognitive-motor dual-tasking elements in pre-game and pre-training routines could be a game-changer for basketball preparation. Coaches and sports scientists should consider implementing cognitive-based warm-ups, especially for athletes who rely on rapid decision-making and reaction-based movements during competition. Practically, cognitive warm-ups should include exercises that mimic real-game scenarios, such as responding to unpredictable stimuli, processing visual cues, and integrating motor and cognitive tasks simultaneously. The use of technology-enhanced drills, such as light-based reaction systems or virtual decision-making simulations, could further optimize preparation and activation before games. Additionally, warm-up protocols should be tailored based on the specific performance goals of a given session—whether focusing on explosive power, agility, or reactive movement speed. In conclusion, modern cognitive warm-ups may represent a promising and practically applicable training tool that can bridge the gap between physical and cognitive readiness in basketball. Their integration into training programs not only enhances immediate performance but may also foster long-term neurophysiological adaptations, leading to improved decision-making, reaction time, and overall athletic performance. Coaches and sports scientists may consider these findings as preliminary support for developing more holistic and scientifically grounded warm-up strategies that address both the physical and cognitive demands of elite sports performance. Overall, this pilot study offers initial support for the relevance of multi-task cognitive warm-ups as a potentially effective preparatory strategy for enhancing specific and unique performance in basketball players.

## Figures and Tables

**Table 1:** Descriptive measurements.

Warm-Up and Tests	N	Mean (s)	SD	Cohen’s d	Cohen’s d	ES Magnitude	CV
T-C-5M	12	1.031	0.028	0.69	0.69	Moderate	0.027
C-C-5M	12	* 1.022	0.025				0.025
T-O-5M	12	1.845	0.119	1	1	Large	0.065
C-O-5M	12	** 1.761	0.094				0.054
T-C-10M	12	1.796	0.057	0.18	0.18	Trivial	0.032
C-C-10M	12	1.793	0.056				0.033
T-O-10M	12	2.656	0.129	1	1	Large	0.049
C-O-10M	12	** 2.535	0.126				0.05
T-O-RT	12	0.757	0.1	1	1	Large	0.133
C-O-RT	12	** 0.683	0.082				0.12

ES: effect size; * Statistically significant difference (*p* < 0.05); ** Statistically significant difference (*p* < 0.01).

## Data Availability

The data presented in this study are available on request from the corresponding author and the first author. The data are not publicly available due to ethical and privacy restrictions.
